# Clinical and allelic heterogeneity in dystrophic epidermolysis bullosa- lessons from an Indian cohort

**DOI:** 10.1371/journal.pone.0289558

**Published:** 2023-08-09

**Authors:** Divya Gupta, Charitha Jayashankar, Manoj Srinivas, Gurudatta Baraka Vishwanathan, Kristipati Raghavendra Reddy, Asha Kubba, Meenakshi Batrani, Ravi Hiremagalore

**Affiliations:** 1 Centre for Human Genetics, Electronic city Phase - I, Bangalore, Karnataka, India; 2 Department of Pediatrics and Dermatology, Manipal Hospital, Bangalore, Karnataka, India; 3 Department of Dermatology, Dr B.R. Ambedkar Medical College and Hospital, Bangalore, Karnataka, India; 4 Delhi Dermpath Laboratory, New Delhi, India; 5 Department of Dermatology, University of Alabama, Birmingham, Alabama, United States of America; Rutgers University Newark, UNITED STATES

## Abstract

**Background:**

Dystrophic epidermolysis bullosa (DEB) is due to variation in the *COL7A1* gene. The clinical phenotype and severity depends on the type of variation and domain of the affected protein.

**Objectives:**

To characterize the spectrum of *COL7A1* variations in a cohort of DEB patients from India, to correlate these findings with clinical phenotypes and to establish a genotype-phenotype correlation.

**Methods:**

This was a retrospective, observational study involving patients with DEB diagnosed on the basis of clinical manifestations, Immuno-fluorescence antigen mapping (IFM) and genetic analysis. A genotype-phenotype correlation was attempted and observations were further explained using IFM on skin biopsies and molecular dynamic simulations. Descriptive statistics were performed using SPSS version 20.0 with P values of <0.05 considered significant.

**Results:**

We report 68 unrelated Indian DEB patients classified as RDEB-Intermediate (RDEB-I), RDEB-Severe (RDEB-S) or DDEB based on the EB diagnostic matrix, immunofluorescence antigen mapping and genetic data. Of 68 DEB patients, 59 (86.76%) were inherited in a recessive pattern (RDEB) and 9 (13.24%) in a dominant pattern (DDEB). Limbal stem cell deficiency was seen in four cases of RDEB-S very early in the course of the disease. A total of 88 variants were detected of which 66 were novel. There were no hotspots and recurrent variations were seen in a very small group of patients. We found a high frequency of compound heterozygotes (CH) in RDEB patients born out of non-consanguineous marriage. RDEB patients older than two years who had oral mucosal involvement, and/or deformities, were more likely to have esophageal involvement. Genotype phenotype correlation showed a higher frequency of extracutaneous manifestations and deformities in patients with Premature Termination Codons (PTCs) than in patients with other variations. Molecular simulation studies in patients with missense mutations showed severe phenotype when they were localized in interrupted regions of GLY-X-Y repeats.

**Conclusion:**

This large study of DEB patients in South Asia adds to the continually expanding genetic database of this condition. This study has direct implications on management as this group of patients can be screened early and managed appropriately.

## Introduction

Epidermolysis bullosa (EB) is a group of inherited blistering disorders with clinical and genetic heterogeneity. There are four major types: EB simplex (EBS), junctional EB (JEB), dystrophic EB (DEB) and mixed EB (Kindler EB) [1,2]. DEB is further classified as dominant DEB (DDEB) and recessive DEB (RDEB) based on the pattern of inheritance. It is caused by variations in *COL7A1*, which encodes type VII collagen. *COL7A1* is one of the largest genes with about 118 exons [3]. While DDEB occurs due to heterozygous glycine substitutions resulting in a milder phenotype, RDEB is usually secondary to substitutions, nonsense, frameshift or splice site variations on both *COL7A1* alleles, which leads to severe disease, with extracutaneous manifestations [4].

Type VII collagen, a major component of anchoring fibrils, has a central triple helical domain (THD) flanked by non-helical domains at each N-terminus end. The THD mainly consists of Glycine–X–Y repeat with interrupted regions [5]. Although over 700 variations in *COL7A1* have been reported to cause DEB, their impact on the structure and function of the protein and on disease pathophysiology are poorly understood [6]. Apart from the genetic make-up, other factors like environment, presence of mutagens, diet and stress also affect the clinical phenotype.

While genotype-phenotype correlations in DEB have been described in various ethnic populations across the world, phenotypic variations amongst these groups continue to be reported [7–11].

One study estimated the incidence and point prevalence of EB to be 41.3 per million live births and 22.4 per million population, respectively. The authors postulated that with a high detection rate in a well-organized set-up, the incidence and prevalence may be higher than previously believed [12]. In South Asia, with over 1 billion population, it is likely that there will be large numbers of EB affected individuals. However so far the attempts to genetically characterize this cohort have been few and far in between. There is no reliable estimate of disease frequency and disease burden from South Asia in literature. With gene therapy completing multiple Phase III clinical trials in the West, there is an urgent need to identify the molecular/ genetic markers of this population. At our centre multidisciplinary EB clinics have been conducted over the past 10 years and patients with different types of EB have been referred for care and management. Here we report the clinical and genetic profile in 68 DEB patients and also attempt a genotype-phenotype correlation in them. Additionally, we have used in-silico modelling of collagen like peptides as surrogate tools to understand the effect of the variations.

## Materials and methods

This was a cross-sectional, retrospective, observational study, involving DEB patients referred to our EB clinic at Centre for Human Genetics and Manipal Hospital, Bangalore, India from 2009 till 2021. Patients were enrolled into the study with informed consent from patients and assent from children where appropriate. The EB matrix was first used to clinically delineate the patients into RDEB-I, RDEB-S and DDEB subgroups, following which the ‘onion-skin approach’ of the consensus criteria (i.e., clinical features, immunofluorescence antigen mapping and exome sequencing) was used to make the final diagnosis [1,2]. Key clinical features, which are specific for DEB, were recorded, viz., mucosal involvement (oral- blisters, ankyloglossia and microstomia; ocular pathology; gastrointestinal tract- esophageal strictures); scarring alopecia of scalp; and deformities (syndactyly and contractures). Esophageal involvement was identified based on a history of dysphagia to solids and liquids and confirmed by barium swallow whenever possible. Ocular involvement was confirmed by a pediatric ophthalmologist with experience in EB. Growth parameters were expressed in adults as BMI and in children as percentiles using the Indian Academy of Pediatrics (IAP) growth charts for boys and girls.

The diagnosis was confirmed by immunofluorescence antigen mapping (IFM) [13] and clinical exome sequencing. The variants identified on exome sequencing, including the novel variants, were confirmed by Sanger validation whenever available, for patients and parents. The effect of variations on the protein functions were predicted using ACMG clinical laboratory standards for next-generation sequencing [14]. All consecutive patients satisfying the above criteria were included for analysis. Patients with absent genetic data were excluded from the study. To understand the prevalence of COL7A1 gene variation, variant allele frequency was calculated as per the following formula: Prevalence of COL7A1 gene variation = number of times of occurrence of COL7A1 gene variation/ total number of exomes scanned.

To understand the effects of missense variations, we performed computational studies of conformation, dynamics and the stability of the protein using the sequence Q02388-collagen alpha-1(VII) chain as the wild type [15]. The parameters included for the simulation were diameter, secondary structure analysis, and hydrogen bond occupancy. The simulation parameters and protocols were as described earlier [16]. The analysis was performed using the gromacs analysis tool and visualized using the PyMol package. (GROMACS version 2020.1- http://www.gromacs.org/ & The PyMOL Molecular Graphics System, Version 2.0 Schrödinger, LLC). The technical conditions and protocols for various genetics methods followed are uploaded in (S1 File).

Patient information was anonymized for the purpose of data analysis. Categorical variables were described using percentages, and continuous variables were described using median and interquartile range. Chi square and Fisher’s exact tests were performed for categorical variables for strength of association. P values of <0.05 were considered as significant. All the analyses were performed using SPSS version 20.0. The study was approved by the Institutional Ethics Committee (Ref No CHG/077/2020-21/001-07/07/2020) and was conducted according to the Helsinki declaration. A written informed consent was obtained from all patients/parents/Guardian. The patients in this manuscript have given written informed consent to publication of their case details.

## Results

### Clinical phenotypes

Sixty eight unrelated Indian DEB patients were enrolled into the study. There were 39 males (57%) and 29 females (43%). Patients were further classified into RDEB-I (27/68, 40%), RDEB-S (29/68, 43%) and DDEB (9/68, 13%) based on the EB diagnostic matrix [17], and the consensus criteria approach [1,2]. Wherever the EB matrix was unable to differentiate between RDEB-I and RDEB-S, the diagnosis was assigned based on presence or absence of certain key features like syndactyly and flexion contractures. In 3/68 patients (4%), the EB matrix provided equal scores for intermediate and severe subtypes.

**Fig 1:**
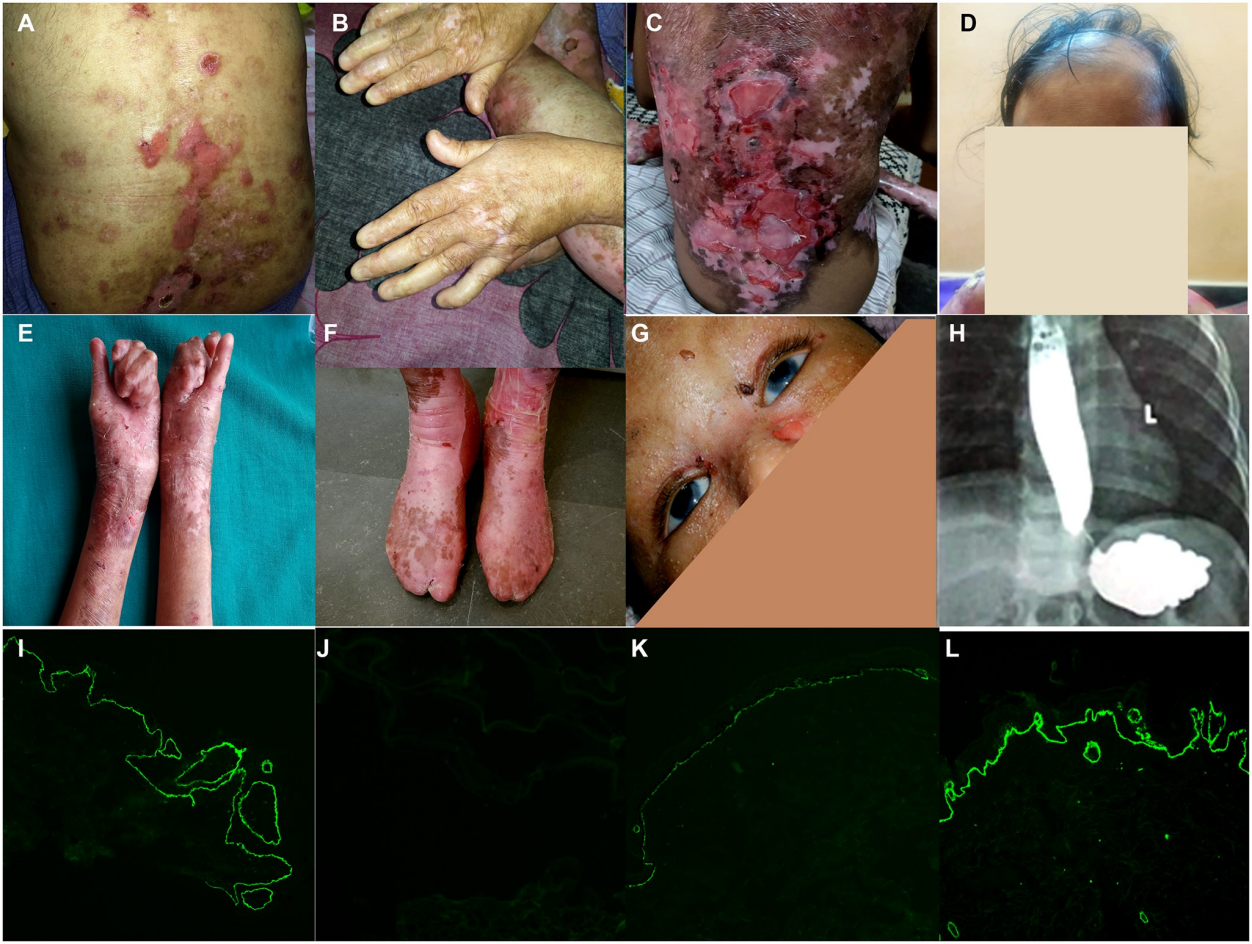
Clinical manifestations and immunofluorescence antigen mapping (IFM) in DEB patients. Clinical manifestations in DEB showing limited skin involvement (A&B) in RDEB-I, extensive skin erosions (C), scarring alopecia (D), deformities (E and F) and limbal stem cell deficiency (G) in RDEB-S. Barium swallow (H) in RDEB showing strictures. IFM on skin biopsy showing normal type VII collagen staining in wild type (10 X) (I), absent (J) in a patient with p.(Arg2745*) (10X), reduced (K) in a patient with compound heterozygous variants p.(Gly400Val) (10X) and p.(Gly2508Asp) and normal (L) staining in a patient with p.(Gly2028Arg) (10X).

The median age of patients was 15 months (IQR 2.75–138.75 months). Only 17 patients were older than 10 years of age. Extracutaneous manifestations including growth failure, esophageal involvement (37/59, 63% in RDEB; 0/9, 0% in DDEB), eye involvement (21/59, 36% in RDEB; 2/9, 22% in DDEB) and webbing of digits and pseudo-syndactyly (29/59, 49% in RDEB; 0/9, 0% in DDEB) were noticed, more commonly in RDEB group as compared to DDEB. The frequency and pattern of clinical involvement in different subtypes of DEB is depicted in Table 1 and Fig 1A–1H. In the RDEB group, the median age of patients who had webbing or fusion was 60 months (IQR 1–185 months) whereas the median age of patients who did not have them was 5 months (IQR 1–90.25 months). Scarring alopecia was seen in 8/59 RDEB patients and limbal stem cell deficiency (LSCD) in four patients. Genito-urinary involvement, photosensitivity or squamous cell carcinoma (SCC) was not detected in any of the patients. Fourteen patients with RDEB died in our cohort, of which definitive age at death was available for 4 patients (10 days, 4 months, 1 year 8 months and 15 years) and estimated age at death was available for another 4 patients (8 years, 9.5 years, 15.5 years, 20 years).

**Table 1:** Frequency of clinical manifestations in the different types of DEB.

	RDEB-S (n = 29)	RDEB-I (n = 27)	RDEB-S/I (n = 3)	DDEB (n = 9)
Gender				
Male	17	17	1	4
Female	12	10	2	5
Age in months				
<2 years	10	18	1	8
>2 years	19	9	2	1
Distribution of wounds				
Limited	0	9	1	2
Generalized	29	18	2	7
Eye involvement				
Present	18	3	0	2
Absent	11	24	3	7
Oral involvement				
Present	29	20	3	1
Absent	0	7	0	8
Esophageal/ GIT involvement				
Present	29	6	2	0
Absent	0	21	1	9
Syndactyly and mitten hand deformity				
Present	26	2	1	0
Absent	3	25	2	9
Scarring alopecia				
Present	7	0	1	0
Absent	22	27	2	9
Consanguinity				
None	12	14	1	8
2^nd^ degree	12	9	1	1
3^rd^ degree	5	2	1	0
4^th^ degree	0	2	0	0

RDEB-S: Recessive Dystrophic Epidermolysis Bullosa-Severe; RDEB-I: Recessive Dystrophic Epidermolysis Bullosa- Intermediate; RDEB-S/I: Recessive Dystrophic Epidermolysis Bullosa cases with overlapping features of Severe and Intermediate type on EB Matrix scoring; DDEB: Dominant Dystrophic Epidermolysis Bullosa.

We noted that 20/27 patients with RDEB-I had oral involvement as opposed to only 1/9 patients with DDEB (p value = 0.001). Out of 68 patients, we also observed that all those over 2 years of age and with oral mucosal involvement also had esophageal involvement while those without oral lesions did not have esophageal involvement either (Spearman correlation coefficient = 0.5; p value = 0.003). Similarly, a higher number of patients over two years of age with deformities had esophageal involvement than those who did not and the difference was statistically significant (Spearman Correlation coefficient = 0.6; p value = 0.000). A positive correlation was noticed between age and development of deformities (Spearman correlation coefficient = 0.3; p value = 0.003) whereas no correlation was noticed between age and development of esophageal involvement (Spearman correlation coefficient = 0.17; p value = 0.16).

### Molecular analysis

**Fig 2:**
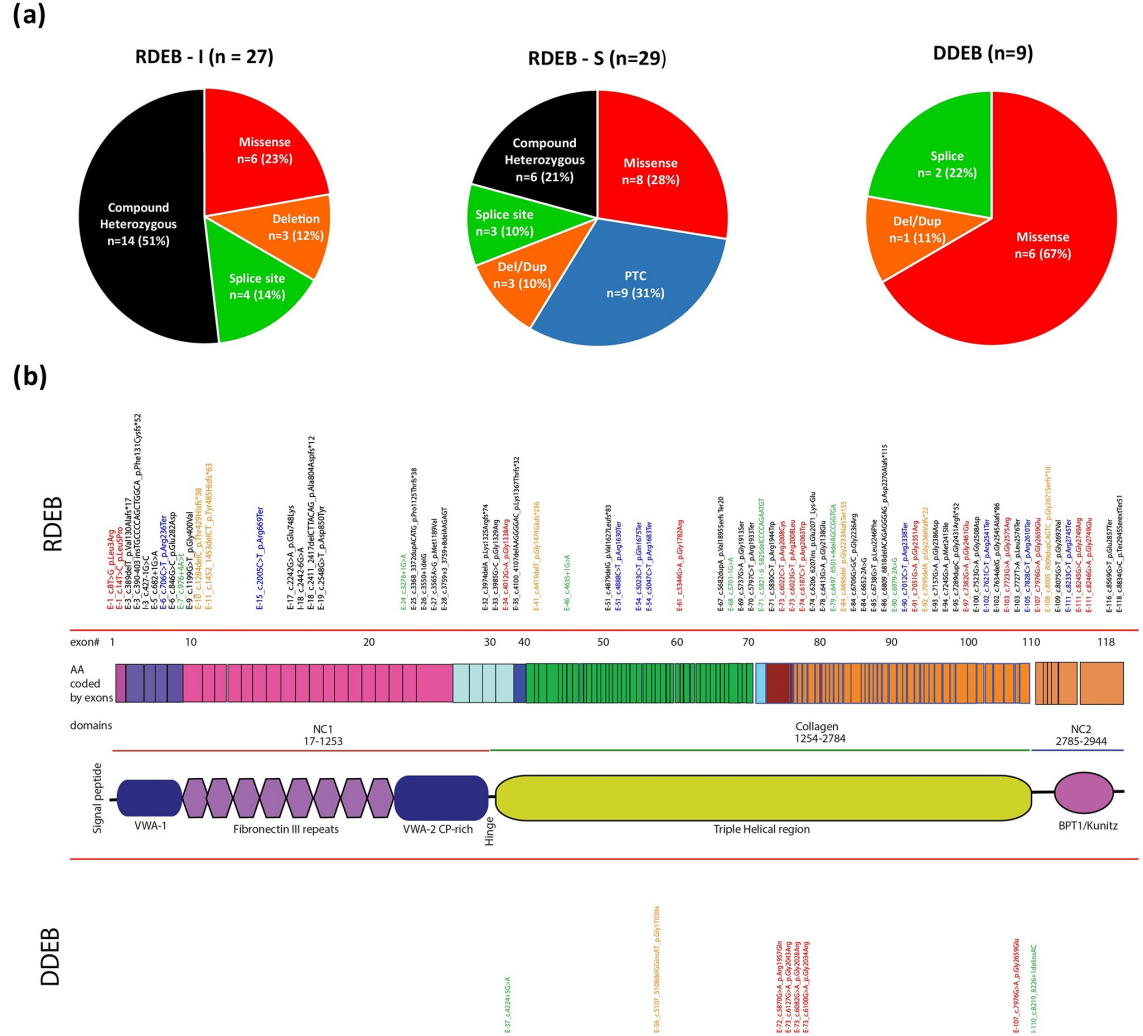
Frequency (2A) and spectrum (2B) of different variations identified in DEB patients. A—Frequency of variations in RDEB-I, RDEB-S & DDEB patients. B—Schematic showing localization of the variants identified in our cohort with exon number, codon change, and amino-acid change mapped to the domains of COL7A1 and color coded as—
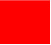
—Missense, 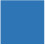
—PTCs (Premature termination codons), 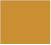
—Deletions/Duplication, 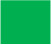
—Splice sites, 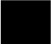
—Compound heterozygous.

Fifty-nine of 68 (86.76%) DEB patients were RDEB and 9 (13.24%) DDEB patients. A total of 88 variants were detected, 79 in RDEB and 9 in DDEB. Of these, 66 variants in 51 patients were novel. Sanger sequencing data from parents were available in 39 variants in 31 patients i.e. we were able to confirm segregation in 31/51 patients with novel variants (S1 Table). The number of recurrent variations in our cohort were only 9 out of the 88 variants (10.22%) and no hotspots were observed. The variations and their frequency in various subtypes of DEB along with the domains involved are shown in Fig 2A and 2B respectively. The compound heterozygous variants were grouped separate from homozygous variants. We also studied the effect of all the 88 variants in our cohort, which were classified according to the ACMG guidelines as ‘pathogenic’, ‘likely pathogenic’ or of ‘uncertain significance’. The detailed description of individual variants in RDEB-I and RDEB-S are presented in Table 2.

**Table 2:** Genotype—Phenotype correlation in DEB subtypes, showing clinical phenotypes, IFM staining patterns, the variations, domains involved, their effect on the protein structure and the novelty status in RDEB-I, RDEB-S, RDEB-S/I and DDEB.

**RDEB–I**																
**Patient**	**A@D**	**Consanguinity**	**Scalp**	**Oral**	**Eye**	**GIT**	**Deformities**	**IFM**	**Zygosity**	**Variation**	**Exon**	**Domain**	**Variant**	**Protein Change**	**ACMG classification**	**PMID**
P1	95	2	No	Yes	No	No	No	NA	Homozygous	MS	Exon 1	NC1	c.14T>C	p.Leu5Pro	Uncertain Significance	Novel
P2	329	2	No	Yes	No	No	No	Normal staining	Homozygous	MS	Exon 61	THD	c.5344G>A	p.Gly1782Arg	Likely Pathogenic	PMID: 8752681
P3	6	2	No	Yes	No	No	No	Normal staining	Homozygous	MS	Exon 73	THD	c.6022C>T	p.Arg2008Cys	Pathogenic	PMID: 29272047
P4	12	0	No	Yes	No	No	No	Normal staining	Homozygous	MS	Exon 73	THD	c.6023G>T	p.Arg2008Leu	Likely Pathogenic	Novel
P5	1	3	No	Yes	No	No	No	NA	Homozygous	MS	Exon 111	THD	c.8245G>C	p.Gly2749Arg	pathogenic	PMID: 8752681
P6	5	2	No	Yes	No	No	No	Normal staining	Homozygous	MS	Exon 111	THD	c.8246G>A	p.Gly2749Glu	Likely pathogenic	Novel
P7	1	0	No	Yes	No	Yes	No	NA	Homozygous	Del	Exon 10	NC1	c.1284delC	p.Thr429Hisfs*38	Likely Pathogenic	Novel
P8	1	3	No	No	No	No	No	Absent staining	Homozygous	Del	Exon 11	NC1	c.1452_1453delCT	p.Tyr485Hisfs*63	Likely Pathogenic	Novel
P9	1	2	No	Yes	No	No	No	Absent staining	Homozygous	Del	Exon 41	THD	c.4419delT	p.Gly1474Glufs*236	Likely Pathogenic	Novel
P10	133	2	No	Yes	Yes	No	No	Absent staining	Homozygous	SS	Exon 7	NC1	c.976+4A>t	nil	Uncertain significace	Novel
P11	47	2	No	No	No	No	Yes	Reduced staining	Homozygous	SS	Exon 24	NC1	c.3276+1G>A	nil	Likely Pathogenic	Novel
P12	1	4	No	Yes	No	Yes	No	Absent staining	Homozygous	SS	Exon 46	THD	c.4635+1G>A	nil	Likely Pathogenic	Novel
P13	1	4	No	No	No	Yes	No	Reduced staining	Homozygous	SS	Exon 90	THD	c.6979-2A>G		Likely Pathogenic	Novel
P14	8	0	No	Yes	No	No	No	NA	Heterozygous	CH-Del	Exon 102	THD	c.7634delG	p.Gly2545Alafs*86	Likely Pathogenic	Novel
									Heterozygous	CH-MS	Exon 109	THD	c.8075G>T	p.Gly2692Val	Likely pathogenic	Novel
P15	7	0	No	No	Yes	No	No	NA	Heterozygous	CH-MS	Exon 6	NC1	c.846G>C	p.Glu282Asp	Likely Pathogenic	PMID: 19681861
									Heterozygous	CH-SS	Intron 18	NC1	c.2442-6G>A	nil	Uncertain Significace	Novel
P16	1	2	No	Yes	No	No	No	Absent staining	Heterozygous	CH-Del	Exon 3	NC1	c.389delT	p.Val130Alafs*17	pathogenic	Novel
									Heterozygous	CH-Ins	Exon 3	NC1	c.390-403 insTGCCCCAGCTGGCA	p.Phe131Cysfs*52	Likely Pathogenic	Novel
P17	1	0	No	Yes	No	Yes	Yes	NA	Heterozygous	CH-Del	Exon 32	THD	c.3974delA	p.Lys1325Argfs*74	likely pathogenic	Novel
									Heterozygous	CH-MS	Exon 78	THD	c.6413G>A	p.Gly2138Glu	likely pathogenic	Novel
P18	193	0	No	Yes	No	No	No	Reduced staining	Heterozygous	CH-MS	Exon 9	NC1	c.1199G>T	p.Gly400Val	Likely pathogenic	Novel
									Heterozygous	CH-MS	Exon 100	THD	c.7523G>A	p.Gly2508Asp	Likely pathogenic	Novel
P19	302	0	No	Yes	Yes	Yes	No	Normal staining	Heterozygous	CH-PTC	Exon 118	NC2	c.8834G>C	p.Ter2945Serext*51	Uncertain significace	Novel
									Heterozygous	CH-SS	Intron 3	NC1	c.427-1G>C	nil	Pathogenic	Novel
P20	3	2	No	Yes	No	No	No	Normal staining	Heterozygous	CH-MS	Exon 27	NC1	c.3565A>G	p.Met1189Val	Uncertain significace	Novel
									Heterozygous	CH-Dup	Exon 95	THD	c.7289dupC	p.Gly2431Argfs*52	Likley pathogenic	Novel
P21	358	0	No	Yes	No	Yes	No	NA	Heterozygous	CH-SS	Exon 26	NC1	c.3550+1delG	nil	Pathogenic	Novel
									Heterozygous	CH-PTC	Exon 70	THD	c.5797C>T	p.Arg1933Ter	Pathogenic	PMID: 27544590
P22	1	0	No	Yes	No	No	No	NA	Heterozygous	CH-Del	Exon 86	THD	c.6809_6818delACAGAGGGAG	p.Asp2270Alafs*115	Pathogenic	Novel
									Heterozygous	CH-SS	Exon 5	NC1	c.682+1G>A	nil	Pathogenic	PMID: 9326325
P23	707	0	No	Yes	No	No	No	NA	Heterozygous	CH-Del	Exon 18	NC1	c.2411_2417delCTTACAG	p.Ala804Aspfs*12	Likely pathogenic	Novel
									Heterozygous	CH-SS	Exon 28	NC1	c.3759+3_3759+8delAAGAGT	nil	Uncertain significance	Novel
P24	5	0	No	Yes	No	No	No	Reduced staining	Heterozygous	CH-PTC	Exon 116	NC2	c.8569G>T	p.Glu2857Ter	Likley pathogenic	Novel
									Heterozygous	CH-MS	Exon 34	THD	c.4012G>A	p.Gly1338Arg	Uncertain significace	PMID: 21448560
P25	2	0	No	No	No	No	No	NA	Heterozygous	CH-MS	Exon 85	THD	c.6738G>T	p.Leu2246Phe	Uncertain significance	Novel
									Heterozygous	CH-MS	Exon 19	NC1	c.2548G>T	p.Asp850Tyr	Uncertain significance	Novel
P26	4	0	No	No	No	No	No	Absent staining	Heterozygous	CH-SS	Exon 84	THD	c.6652-2A>G	nil	Likley pathogenic	Novel
									Heterozygous	CH-MS	Exon 71	THD	c.5830C>T	p.Arg1944Trp	Uncertain significace	Novel
P27	432	0	No	No	No	No	No	NA	Heterozygous	CH-Ins	Exon 74	THD	c.6206_6207insGGGAGAGAAAGTAGAACG	p.Glu2071_Gln2072insLys-Glu	Unknown significace	Novel
									Heterozygous	CH-MS	Exon 17	NC1	c.2242G>A	p.Glu748Lys	Unknown significace	Novel
**RDEB–S**																
P28	6	2	No	Yes	No	Yes	Yes	NA	Homozygous	MS	Exon 1	NC1	c.8T>G	p.Leu3Arg	Uncertain significance	Novel
P29	22	2	No	Yes	No	Yes	No	NA	Homozygous	MS	Exon 34	THD	c.4012G>A	p.Gly1338Arg	Uncertain signifcance	PMID: 19665875
P30	372	3	No	Yes	No	Yes	Yes	NA	Homozygous	MS	Exon 91	THD	c.7051G>A	p.Gly2351Arg	Likely pathogenic	PMID: 8752681
P31	3	2	No	Yes	Yes	Yes	Yes	Reduced staining	Homozygous	MS	Exon 97	THD	c.7382G>A	p.Gly2461Glu	Likley Pathogneic	Novel
P32	46	0	Yes	Yes	Yes	Yes	Yes	Reduced staining	Homozygous	MS	Exon 103	THD	c.7723G>A	p.Gly2575Arg	Pathogenic	PMID: 8592061
P33	156	3	No	Yes	Yes	Yes	Yes	NA	Homozygous	MS	Exon 107	THD	c.7976G>A	p.Gly2659Glu	Likely Pathogenic	Novel
P34	251	0	Yes	Yes	Yes	Yes	Yes	Normal staining	Homozygous	MS	Exon 111	THD	c.8246G>A	p.Gly2749Glu	Uncertain significance- likely damaging	Novel
P35	231	0	Yes	Yes	Yes	Yes	Yes	Normal staining	Homozygous	MS	Exon 111	THD	c.8246G>A	p.Gly2749Glu	Uncertain significance- likely damaging	Novel
P36	1	3	No	Yes	No	Yes	Yes	Absent staining	Homozygous	PTC	Exon 6	NC1	c.706C>T	p.Arg236Ter	Pathogenic	PMID: 8037207
P37	60	0	No	Yes	Yes	Yes	Yes	Absent staining	Homozygous	PTC	Exon 15	NC1	c.2005C>T	p.Arg669Ter	pathogenic	PMID: 9881948
P38	45	2	No	Yes	Yes	Yes	Yes	Absent staining	Homozygous	PTC	Exon 51	THD	c.4888C>T	p.Arg1630Ter	pathogenic	PMID: 10367729
P39	9	0	Yes	Yes	Yes	Yes	Yes	Reduced staining	Homozygous	PTC	Exon 54	THD	c.5023C>T	p.Gln1675Ter	Likley Pathogenic	Novel
P40	12	2	No	Yes	No	Yes	Yes	Absent staining	Homozygous	PTC	Exon 54	THD	c.5047C>T	p.Arg1683Ter	pathogenic	PMID: 16189623,PMID: 21448560
P41	185	2	No	Yes	Yes	Yes	Yes	NA	Homozygous	PTC	Exon 54	THD	c.5047C>T	p.Arg1683Ter	Pathogenic	PMID: 16189623,PMID: 21448560
P42	68	3	No	Yes	No	Yes	Yes	NA	Homozygous	PTC	Exon 102	THD	c.7621C>T	p.Arg2541Ter	Likley pathogenic	PMID: 19681861
P43	2	3	No	Yes	Yes	Yes	Yes	Absent staining	Homozygous	PTC	Exon 105	THD	c.7828C>T	p.Arg2610Ter	Pathogenic	PMID: 9326325
P44	100	2	Yes	Yes	Yes	Yes	Yes	Absent staining	Homozygous	PTC	Exon 111	THD	c.8233C>T	p.Arg2745Ter	Pathogenic	PMID: 19681861
P45	256	2	No	Yes	Yes	Yes	Yes	NA	Homozygous	Del	Exon 84	THD	c.6696del	p.Gly2233Alafs*155	Pathogenic	Novel
P46	201	0	No	Yes	No	Yes	Yes	NA	Homozygous	Del	Exon 92	THD	c.7095delA	p.Gly2366Valfs*22	Likely pathogenic	Novel
P47	57	2	No	Yes	Yes	Yes	Yes	NA	Homozygous	Dup	Exon 108	THD	c.8005_8009dupCAGTC	p.Gly2671Serfs*10	Likely Pathogenic	Novel
P48	25	2	No	Yes	Yes	Yes	Yes	Absent staining	Homozygous	SS	Exon 68	THD	c.5701-1G>A	nil	Pathogenic	Novel
P49	170	2	Yes	Yes	Yes	Yes	Yes	Reduced staining	Homozygous	SS	Exon 71	THD	c.5821- 6_5825delCCCCAGAATGT	nil	Likely pathogenic	Novel
P50	3	2	No	Yes	Yes	Yes	No	Absent staining	Homozygous	SS	Exon 79	THD	c.6497_6501+4delAGCCGGTGA	nil	Likley Pathogenic	Novel
P51	100	0	No	Yes	No	Yes	Yes	Absent staining	Heterozygous	CH-MS	Exon 94	THD	c.7245G>A	p.Met2415Ile	Pathogenic	PMID: 16470588
									Heterozygous	CH-PTC	Exon 103	THD	c.7727T>A	p.Leu2576Ter	Likley Pathogenic	Novel
P52	1	0	No	Yes	No	Yes	No	Reduced staining	Heterozygous	CH-Dup	Exon 25	NC1	c.3368_3372dupACATG	p.Pro1125Thrfs*38	Likely patogenic	Novel
									Heterozygous	CH-Del	Exon 35	THD	c.4100_4107delAAGGGGAC	p.Lys1367Thrfs*32	Like;ly pathogenic	Novel
P53	85	0	No	Yes	Yes	Yes	Yes	NA	Heterozygous	CH-Dup	Exon 67	THD	c.5682dupA	p.Val1895Serfs Ter20	Likely pathogenic	Novel
									Heterozygous	CH-MS	Exon 84	THD	c.6706G>C	p.Gly2236Arg	Likely Pathogenic	Novel
P54	220	0	Yes	Yes	No	Yes	Yes	NA	Heterozygous	CH-Del	Exon 51	THD	c.4879delG	p.Val1627Leufs*83	Likely pathogenic	Novel
									Heterozygous	CH-MS	Exon 69	THD	c.5737G>A	p.Gly1913Ser	Uncertain significace	Novel
P55	19	0	No	Yes	Yes	Yes	Yes	Reduced staining	Heterozygous	CH-MS	Exon 93	THD	c.7157G>A	p.Gly2386Asp	Uncertain significace	Novel
									Heterozygous	CH-Del	Exon 32	THD	c.3974delA	p.Lys1325Argfs*74	Likley Pathogenic	Novel
P56	32	0	No	Yes	No	Yes	Yes	Normal staining	Heterozygous	CH-MS	Exon 33	THD	c.3985G>C	p.Gly1329Arg	not available (need to check)	Novel
									Heterozygous	CH-MS	Exon 17	NC1	c.2242G>A	p.Glu748Lys	Unknown significance	Novel
**RDEB–S/I**																
P57	347	2	Yes	Yes	No	No	Yes	NA	Homozygous	MS	Exon 73	THD	c.6023G>T	p.Arg2008Leu	Likley pathogenic	Novel
P58	76	0	No	Yes	No	Yes	No	Normal staining	Homozygous	MS	Exon 74	THD	c.6187C>T	p.Arg2063Trp	Pathogenic	Novel
P59	1	3	No	Yes	No	Yes	No	NA	Homozygous	PTC	Exon 90	THD	c.7012C>T	p.Arg2338Ter	Pathogenic	PMID: 10367729
**DDEB**																
P60	11	0	No	Yes	Yes	No	No	NA	Heterozygous	MS	Exon 72	THD	c.5870G>A	p.Arg1957Gln	Uncertain significance	Novel
P61	2	0	No	No	No	No	No	NA	Heterozygous	MS	Exon 73	THD	c.6127G>A	p.Gly2043Arg	Likely pathogenic	Novel
P62	11	0	No	No	No	No	No	Normal staining	Heterozygous	MS	Exon 73	THD	c.6082G>A	p.Gly2028Arg	Pathogenic	PMID: 10836608
P63	1	0	No	No	No	No	No	Normal staining	Heterozygous	MS	Exon 73	THD	c.6100G>A	p.Gly2034Arg	Likely pathogenic	PMID: 9347800
P64	1	2	No	No	No	No	No	Absent staining	Heterozygous	MS	Exon 107	THD	c.7976G>A	p.Gly2659Glu	Likley pathogenic	Novel
P65	6	0	No	No	No	No	No	Normal staining	Heterozygous	MS	Exon 107	THD	c.7976G>A	p.Gly2659Glu	likley pathogenic Likley Pathogenic	Novel
P66	17	0	No	No	No	No	No	Normal staining	Heterozygous	Del	Exon 56	THD	c.5107_5108delGGinsAT	p.Gly1703Ile	Likely pathogenic	Novel
P67	13	0	No	No	No	No	No	Normal staining	Heterozygous	SS	Exon 37	THD	c.4224+5G>A	nil	Variant of uncertain significance	Novel
P68	293	0	No	No	Yes	No	No	NA	Heterozygous	SS	Intron 110	THD	c.8219_8226+1delinsAC	nil	Variant of uncertain significance	Novel

**Fig 3:**
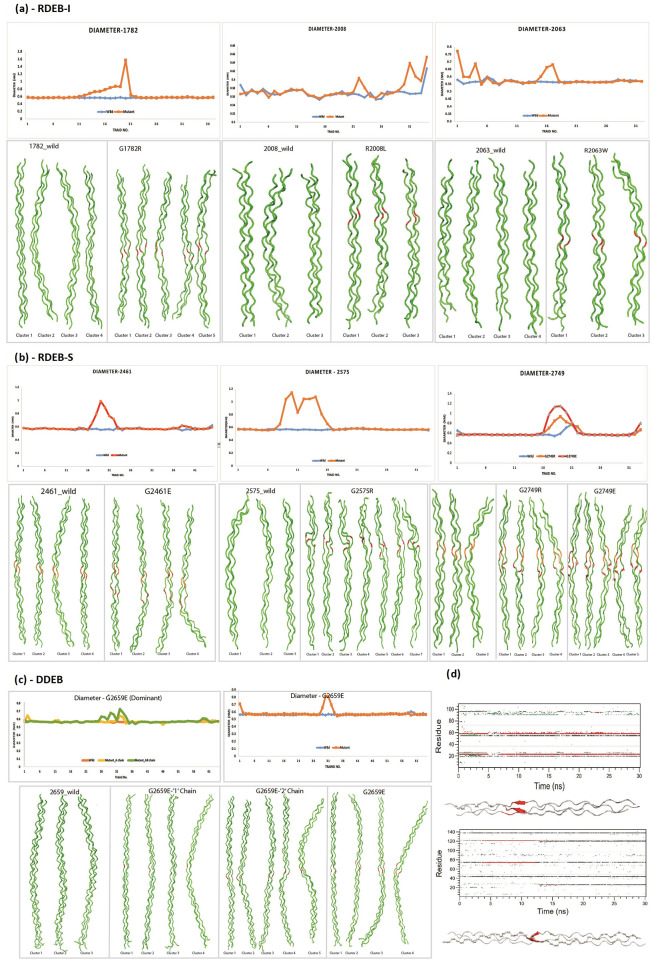
Molecular dynamic simulations in DEB subtypes. A and B—Diameter analysis and illustrative structure of the clusters for wild type and missense variations for RDEB-I & RDEB-S subtypes. C—Showing the changes in Diameter & Cluster analysis in wild type, 1 chain, 2 chains and all 3 chains of collagen 7 substitution model of G2659E. D—Showing the formation of beta sheets in the substitution models G2749R & G2461E of the RDEB-S subtype.

We also studied the effect of glycine and non-glycine substitutions in 3 RDEB-I, 4 RDEB-S & 1 DDEB using molecular dynamic simulations (Fig 3 and Table 3). In RDEB-S, simulations were performed in missense variants G2749R, G2749E, G2461E, G2575R and in RDEB-I for G1782R, R2008L and R2063W. We also performed the simulation for G2659E which was inherited both in autosomal recessive (AR) and dominant (AD) patterns. All simulations involving substitutions were compared with the respective wild-type amino acid sequence. The diameter of the wild type ranged from 0.5–0.7nm while the mutant protein ranged from 0.6–1.1nm. The increase in diameter was higher in mutants involving the interrupted region and/or in those where glycine was substituted by arginine or glutamine. In the G2659E model, changes in diameter were more pronounced in the AR than AD pattern. With respect to changes in secondary structure, formation of beta sheets was seen in G2749R and G2461E mutants, both of which were present in the interrupted region. In the first case, the changes were persistent throughout the simulation while in the latter it was transient. This change was reflected by changes in the dihedral angles (phi and psi) in both models, when compared to wild types. Finally, in all mutants, there was a reduction in hydrogen bond occupancy that was more pronounced in glycine substitutions than in non-glycine substitutions. Hydrogen bond occupancy indirectly reflects the stability between the three chains of Type VII collagen and typically occurs between the glycine of one chain with X of the other chain in the G-X-Y repeats.

**Table 3:** (A,B,C) showing parameters used in molecular dynamic simulations in RDEB-I, RDEB-S and DDEB subtypes respectively.

**A. RDEB–I**							
Model	1782		2008		2063		
Mutation	Wild	G1782R	Wild	G2008L	Wild	G2063W	
IFM		Normal		Normal		Normal	
Interruption	Absent	Absent	Absent	Absent	Absent	Absent	
Diameter at Mutation site (nm)	0.552	0.86651	0.56439	0.60311	0.563013	0.68019	
Helix-Beta transition	NA	NA	NA	NA	NA	NA	
Phi Angle of substituted residue	NA	NA	NA	NA	NA	NA	
Psi angle of substituted residue	NA	NA	NA	NA	NA	NA	
H-Bond Occupancy (in %)	58.66	45.56	56.18	54.22	48.18	42.70	
**B. RDEB–S**							
Model	2461		2575		2749		
Mutation	Wild	G2461E	Wild	G2757R	Wild	G2749R	G2749E
IFM		Normal		Reduced		No IFM	Normal
Interruption	Present	Present	Absent	Absent	Present	Present	Present
Diameter at Mutation site (nm)	0.553201	0.9854	0.560882	0.8286	0.7115	0.93487	1.14542
Helix-Beta transition	Transient	Transient	NA	NA	Transient	Persistent	NA
Phi Angle of substituted residue	-63.44	-97.82	NA	NA	-67.59	-141.368	-61.39
Psi angle of substituted residue	140.4	141.2	NA	NA	83.33	133.7	150.71
H-Bond Occupancy	56.69%	46.53%	57.10%	45.11%	55.34%	49.18%	50.14%
**C. DDEB**							
Model	2659						
Mutation	Wild		’1’ Chain		’2’ Chain	’all’ Chains	
IFM			Absent		Absent	NA	
Interruption	Absent		Absent		Absent	Absent	
Diameter at Mutation site (nm)	0.6		0.643		0.723	0.8111	
Helix-Beta transition	NA		NA		NA	NA	
Phi Angle of substituted residue	NA		NA		NA	NA	
Psi angle of substituted residue	NA		NA		NA	NA	
H-Bond Occupancy	54.83%		49.67%		55.92%	51.83%	

IFM: Immunofluorescence mapping; NA: Not Applicable.

### Genotype-phenotype correlation

The detailed clinical phenotypes, IFM, genetic variations and its in-silico predictions and the domains of the protein affected in 59 RDEB and 9 DDEB patients are shown in Table 2.

Overall, PTCs were more likely to be associated with severe or RDEB-S phenotype (9/10, 90%) and compound heterozygous variations were more likely to be associated with a milder or RDEB-I phenotype (14/20, 70%). In RDEB patients, those with PTCs showed a higher frequency of extracutaneous manifestations than patients with other variations. Specifically, all patients with PTCs (10/10, 100%) had esophageal involvement whereas 9/16, 4/6, 5/7 and 9/20 patients with missense, del/dup, splice site and CH variations respectively, had esophageal involvement. Similarly, 9/10, 8/16, 3/6, 3/7 and 6/20 patients with PTC, missense, del/dup, splice site and CH variations respectively, had syndactyly and mitten hand deformity. The difference between the proportion of PTCs compared with the proportion of all other variations that were associated with esophageal involvement and hand deformities was statistically significant (p value = 0.004 and 0.0004 respectively).

A higher number of patients with extracutaneous manifestations had variations localized to THD than NC1 (27/35; 77.14% Vs 7/35; 20%; p = 0.115). Specifically, esophageal involvement (26/37; 70.27% vs. 3/37; 8.1%; p = 0.007) and deformities (19/29; 65.5% vs 4/29; 13.8%; p value = 0.142) were observed in a higher number of patients with THD variations than those localized to NC1 domain. The difference was statistically significant only with respect to esophageal involvement.

Of the 59 RDEB patients, 32 (54%) had a family history of consanguinity, 13 with RDEB-I, 17 with RDEB-S and 2 with RDEB-I/S. We observed that out of 20 compound heterozygotes in our cohort, 18 patients (90%) did not have any history of consanguinity in their parents. This prompted us to study the allele frequency of collagen VII variants in Indian population and we were able to get data for four variants (S2 Table).

In RDEB-I patients, 2 each with deletion (66.6%), splice site (50%) and compound heterozygous (14.2%) variations showed absent Type VII collagen staining. Two patients each with splice site (50%) and compound heterozygous (14.2%) variation showed reduced staining. Whereas, 4 patients with missense (66.6%) and 2 compound heterozygous (14.2%) variations showed normal staining.

In RDEB-S patients, 6 with PTCs (66.6%), 2 splice sites (66.6%) and 1 compound heterozygous (16.6%) variation showed absent Type VII collagen staining. Two each with missense (25%) and compound heterozygous (33.3%) and one each with PTCs (11.1%) and splice site (33.3%) variation showed reduced staining. Two with missense (25%) & 1 compound heterozygous (16.6%) variation showed normal staining.

## Discussion

We describe the clinical subtypes, age at presentation and the spectrum of extracutaneous features in 68 Indian DEB patients from unrelated families. Our observations suggest that children older than 2 years of age, and with oral mucosal involvement and/or deformities were more likely to have esophageal involvement than those without them. This difference was statistically significant (p value = 0.003 and 0.000 respectively). Hence, it would be appropriate to investigate RDEB patients with oral mucosal involvement and deformities with concomitant swallowing difficulty with a barium swallow to rule out esophageal strictures.

It was interesting to note that the majority of patients with RDEB-I type had oral involvement as opposed to DDEB where it was absent. This finding was statistically significant and may be a clinical clue to differentiate milder forms of RDEB from DDEB. Certain clinical manifestations including scalp involvement and squamous cell carcinoma (SCC) were either seen less frequently or not seen at all (8/59 RDEB cases of scalp involvement and no cases of SCC) in our cohort. This can possibly be attributed to the fact that the majority of the patients in our cohort were less than 5 years of age (36/59 patients). Since scarring complications in DEB evolve over time, it is likely that the younger age of our cohort skewed the results towards a lower frequency of these manifestations.

We also report 4 patients with LSCD in RDEB-S and this was not associated with any specific type of variation. Vazirani et al and Cheung et al have reported EB as one of the causes of LSCD [18,19]. Previously Thanos et al have described LSCD in an 11.5-year-old boy with RDEB-S, which was successfully treated with human amniotic membrane [20]. All patients in our study also had generalized severe type of RDEB, and it may be worthwhile for the ophthalmologist to specifically look for this complication in such cases. It could be speculated that a chronic inflammatory state in the cornea in RDEB could result in this condition [21]. Larger studies supported by mouse models would help understand the condition better.

Our study confirms that DDEB is a consequence of glycine substitutions, a majority localized to the THD although del/dup and splice-site variants were also observed.

Our study had a relatively higher percentage of compound heterozygous variations (20/59; 33.89%) which came from the non-consanguineous parents (18/20, 90%). This is a significant finding and might indicate a high frequency of collagen VII mutations in our population.

Universally, RDEB-S is a result of nonsense, missense, deletions/duplications and splice site variations, the majority being localized in the THD [4,9,11,22]. Our observations confirm that nonsense variations with PTCs contributing to 30% of cases and splice-site variations, del/dup in about 10% each, all of which behaved like PTCs resulting in a premature truncation of the amino acid chain, a shortened non-functional protein and consequently absent type VII collagen staining on IFM and a severe phenotype. Missense and compound heterozygous variations contributed to almost half the RDEB-S cases and were mostly localized to the THD. THD contains Gly-X-Y repeats that are interrupted in certain areas by non-glycine residues. We attempted to explain the severity of phenotype in missense variations through molecular simulations [23]. We observed changes in diameter and secondary structure more pronounced in variations occurring in the interrupted regions and a loss of conventional hydrogen bonds, resulting in increased diameter of the triple helix. These can be explained by glycine substitutions with bulky amino acids like arginine and glutamine [24]. Taken together, these changes may reduce the ability to form trimers leading to the formation of an abnormal type VII collagen or make it more susceptible to extracellular matrix proteases, finally resulting in compromised integrity of the anchoring fibrils and hence a more severe phenotype [24–28]. However, these cases demonstrated partial type VII collagen assembly which was evidenced by a positive staining for the same on IFM. Also, the molecular simulations were performed on hypothetical peptide models and hence electron microscopic studies or functional assays may be necessary to validate these simulations. Six compound heterozygous patients in RDEB-S were a combination of missense, PTC, deletion and duplication variations (Table 2). Most were localized to the THD and were eventually behaving like a PTC resulting in a severe phenotype. This was identified as an absent or reduced staining for type VII collagen on IFM and the appearance of extensive scarring and deformities in these patients.

Our study confirms that bi-allelic missense variations or in combination with either a PTC or del/dup or splice site variation could result in RDEB-I. Most of the variations were localized to either NC1 or NC2 or a combination with THD. Out of 27 patients with RDEB-I subtype, the majority had compound heterozygous (n = 14) and missense (n = 6) variations, where there was either no loss of protein or only a minor alteration of the protein. The simulation performed on the 3 RDEB-I variants also revealed that there were minor changes in the diameter and hydrogen bond occupancy, no changes were seen in secondary structures and these substitutions were away from the interruptions of the Gly-X-Y repeats all leading to a milder phenotype. This was reflected as normal or reduced type VII collagen staining on IFM. Clinically, these patients had only cutaneous involvement or very limited extracutaneous involvement. We observed that some of the patients with splice-site variations showed a milder RDEB-I phenotype. However they were mostly less than 2 years of age and a longer follow-up would help ascertain whether these patients developed more severe features.

Several studies have described genotype-phenotype correlations in DEB [3,7,8,10,11,22,29–34]. We noted that there was a significant association between PTCs and mitten hand deformity and esophageal involvement, as compared with all other mutations combined. Similarly, mutations localized to the THD were more likely to be associated with esophageal involvement. This has implications with respect to clinical care as these children can be identified in a timely fashion and be followed up more often. More funds can be earmarked early on for them, thus enabling a more efficient utilization of resources in resource-limited settings.

This study, being a retrospective study, many of our older patients were lost to follow-up or had expired. Hence clinical phenotyping could not be up-to-date with features developing over time. Immunofluorescence antigen mapping was performed only in cases where a clinical diagnosis could not be established. Hence the expression of the mutant protein in the skin was not possible in all the patients, which would have helped in understanding the disease mechanism better. We did not use any scoring systems to assess severity. Instead, the EB diagnostic matrix was used to clinically classify DEB patients, the accuracy of which is limited below 2 years of age. Larger prospective study of DEB patients with complete clinical phenotyping would help in better genotype-phenotype correlation. This may also have implications in choosing the right candidate for gene therapy when it becomes a possibility.

## Conclusion

This is a single-centre study representing a large cohort of DEB patients from South Asia, including clinical and genetic data. RDEB patients above 2 years of age with oral involvement and/or deformities were noted to have a higher frequency of esophageal involvement thus emphasizing the need for a barium swallow early in the course of the disease. Unusual manifestations in the cohort included LSCD. The study reports a higher number of patients with compound heterozygous variations indicating the prevalence of such alleles in the society. A combination of molecular simulations and IFM was used to explain severity that is fairly consistent with existing knowledge. A large number of novel mutations, and absent hotspots reflect the need to individualize genetic profiles in different ethnic groups for appropriate genetic counselling, prenatal diagnosis and gene therapy.

## Supporting information

S1 Checklist STROBE Statement—Checklist of items that should be included in reports of observational studies. (DOCX)

S1 Table Parental genotype data in novel variants. (XLSX)

S2 Table Allele frequency in four CH variants. (DOCX)

S1 File Protocols. (DOCX)

## Abbreviations

EB: Epidermolysis bullosa
DEB: Dystrophic Epidermolysis Bullosa
RDEB: Recessive Dystrophic Epidermolysis Bullosa
RDEB - I: RDEB intermediate
RDEB - S: RDEB severe
DDEB: Dominant Dystrophic Epidermolysis Bullosa
IFM: Immunofluorescence mapping
LSCD: Limbal stem cell deficiency
BMI: Body Mass Index
IAP: Indian Academy of Pediatrics
SCC: Squamous cell carcinoma
AR: Autosomal Recessive
AD: Autosomal Dominant
CH: Compound Heterozygous
Del & Dup: Deletions & Duplications
PTCs: Premature termination codons
THD: Triple Helical Domain
