# Supplementary material for: Clinical and allelic heterogeneity in dystrophic epidermolysis bullosa- lessons from an Indian cohort
Source: PLoS One. 2023 Aug 9;18(8):e0289558. doi: 10.1371/journal.pone.0289558 (PMC10411825; doi:10.1371/journal.pone.0289558)
Supplement: S1 Checklist — (DOCX) [file pone.0289558.s001.docx]

STROBE Statement—checklist of items that should be included in reports of observational studies

|  | Item No. | Recommendation | Page  No. | Relevant text from manuscript |
| --- | --- | --- | --- | --- |
| **Title and abstract** | 1 | (*a*) Indicate the study’s design with a commonly used term in the title or the abstract | 1 | Title and Authors |
|  |  | (*b*) Provide in the abstract an informative and balanced summary of what was done and what was found | 4.5 | Abstract |
| Introduction | | | |  |
| Background/rationale | 2 | Explain the scientific background and rationale for the investigation being reported | 6 | Introduction |
| Objectives | 3 | State specific objectives, including any prespecified hypotheses | 6 | clinical and genetic profile in 68 DEB patients and also attempt a genotype-phenotype correlation |
| Methods | | | |  |
| Study design | 4 | Present key elements of study design early in the paper | 7 | cross-sectional observational study |
| Setting | 5 | Describe the setting, locations, and relevant dates, including periods of recruitment, exposure, follow-up, and data collection | 7 | referred to our EB clinic from 2009 till 2021 |
| Participants | 6 | (*a*) *Cohort study*—Give the eligibility criteria, and the sources and methods of selection of participants. Describe methods of follow-up  *Case-control study*—Give the eligibility criteria, and the sources and methods of case ascertainment and control selection. Give the rationale for the choice of cases and controls  *Cross-sectional study*—Give the eligibility criteria, and the sources and methods of selection of participants | 7 | DEB patients referred to our EB clinic from 2009 till 2021. Patients were classified into the DEB subtypes based on the consensus criteria |
|  |  | (*b*) *Cohort study*—For matched studies, give matching criteria and number of exposed and unexposed  *Case-control study*—For matched studies, give matching criteria and the number of controls per case | NA |  |
| Variables | 7 | Clearly define all outcomes, exposures, predictors, potential confounders, and effect modifiers. Give diagnostic criteria, if applicable | 7 | Patients were classified into the DEB subtypes based on the consensus criteria.^1, 2^ Esophageal involvement was identified based on a history of dysphagia to solids and liquids and confirmed by barium swallow whenever possible. Ocular involvement was confirmed by a pediatric ophthalmologist with experience in EB. Growth parameters were expressed in adults as BMI and in children as percentiles using the Indian Academy of Pediatrics (IAP) growth charts for boys and girls.  The diagnosis was confirmed by immunofluorescence antigen mapping (IFM)^13^ and clinical exome sequencing |
| Data sources/ measurement | 8* | For each variable of interest, give sources of data and details of methods of assessment (measurement). Describe comparability of assessment methods if there is more than one group | 7 | Same as above in 7 |
| Bias | 9 | Describe any efforts to address potential sources of bias | NA |  |
| Study size | 10 | Explain how the study size was arrived at | 7 | referred to our EB clinic from 2009 till 2021 |

Continued on next page

| Quantitative variables | 11 | Explain how quantitative variables were handled in the analyses. If applicable, describe which groupings were chosen and why | NA |  |
| --- | --- | --- | --- | --- |
| Statistical methods | 12 | (*a*) Describe all statistical methods, including those used to control for confounding | 7 | Categorical variables were described using percentages, and continuous variables were described using median and interquartile range. Chi square and Fisher's exact tests were performed for categorical variables for strength of association. P values of <0.05 were considered as significant. All the analyses were performed using SPSS version 20.0. |
|  |  | (*b*) Describe any methods used to examine subgroups and interactions | NA |  |
|  |  | (*c*) Explain how missing data were addressed | NA |  |
|  |  | (*d*) *Cohort study*—If applicable, explain how loss to follow-up was addressed  *Case-control study*—If applicable, explain how matching of cases and controls was addressed  *Cross-sectional study*—If applicable, describe analytical methods taking account of sampling strategy | NA |  |
|  |  | (*e*) Describe any sensitivity analyses | NA |  |
| Results | | | | |
| Participants | 13* | (a) Report numbers of individuals at each stage of study—eg numbers potentially eligible, examined for eligibility, confirmed eligible, included in the study, completing follow-up, and analysed | NA |  |
|  |  | (b) Give reasons for non-participation at each stage | NA |  |
|  |  | (c) Consider use of a flow diagram | NA |  |
| Descriptive data | 14* | (a) Give characteristics of study participants (eg demographic, clinical, social) and information on exposures and potential confounders | NA |  |
|  |  | (b) Indicate number of participants with missing data for each variable of interest | NA |  |
|  |  | (c) *Cohort study*—Summarise follow-up time (eg, average and total amount) | NA |  |
| Outcome data | 15* | *Cohort study*—Report numbers of outcome events or summary measures over time |  |  |
|  |  | *Case-control study—*Report numbers in each exposure category, or summary measures of exposure |  |  |
|  |  | *Cross-sectional study—*Report numbers of outcome events or summary measures | 8-14 | All tables n figures |
| Main results | 16 | (*a*) Give unadjusted estimates and, if applicable, confounder-adjusted estimates and their precision (eg, 95% confidence interval). Make clear which confounders were adjusted for and why they were included | 8-14 |  |
|  |  | (*b*) Report category boundaries when continuous variables were categorized | NA |  |
|  |  | (*c*) If relevant, consider translating estimates of relative risk into absolute risk for a meaningful time period | NA |  |

Continued on next page

| Other analyses | 17 | Report other analyses done—eg analyses of subgroups and interactions, and sensitivity analyses | NA |  |
| --- | --- | --- | --- | --- |
| Discussion | | | | |
| Key results | 18 | Summarise key results with reference to study objectives | 15-18 | See text |
| Limitations | 19 | Discuss limitations of the study, taking into account sources of potential bias or imprecision. Discuss both direction and magnitude of any potential bias | 18 | This study, being a retrospective study, many of our older patients were lost to follow-up or had expired. Hence clinical phenotyping could not be up-to-date with features developing over time. Immunofluorescence antigen mapping was performed only in cases where a clinical diagnosis could not be established. Hence the expression of the mutant protein in the skin was not possible in all the patients, which would have helped in understanding the disease mechanism better. We did not use any scoring systems to assess severity. Instead, the EB diagnostic matrix was used to classify DEB patients, the accuracy of which is limited below 2 years of age. |
| Interpretation | 20 | Give a cautious overall interpretation of results considering objectives, limitations, multiplicity of analyses, results from similar studies, and other relevant evidence | 18 | See conclusions |
| Generalisability | 21 | Discuss the generalisability (external validity) of the study results | 18 | Larger prospective study of DEB patients with complete clinical phenotyping would help in better genotype-phenotype correlation. This may also have implications in choosing the right candidate for gene therapy when it becomes a possibility. A large number of novel mutations, and absent hotspots reflect the need to individualize genetic profiles in different ethnic groups for appropriate genetic counselling, prenatal diagnosis and gene therapy |
| Other information | |  | | |
| Funding | 22 | Give the source of funding and the role of the funders for the present study and, if applicable, for the original study on which the present article is based | 2 | Title page, Epidermolysis Bullosa Research Partnership (EBRP); The Government of Karnataka; Centre for Human Genetics, Karnataka.India. |

*Give information separately for cases and controls in case-control studies and, if applicable, for exposed and unexposed groups in cohort and cross-sectional studies.

**Note:** An Explanation and Elaboration article discusses each checklist item and gives methodological background and published examples of transparent reporting. The STROBE checklist is best used in conjunction with this article (freely available on the Web sites of PLoS Medicine at http://www.plosmedicine.org/, Annals of Internal Medicine at http://www.annals.org/, and Epidemiology at http://www.epidem.com/). Information on the STROBE Initiative is available at www.strobe-statement.org.
