# Supplementary material for: Clinical and allelic heterogeneity in dystrophic epidermolysis bullosa- lessons from an Indian cohort
Source: PLoS One. 2023 Aug 9;18(8):e0289558. doi: 10.1371/journal.pone.0289558 (PMC10411825; doi:10.1371/journal.pone.0289558)
Supplement: S2 Table — (DOCX) [file pone.0289558.s003.docx]

| SL NO | VARIANT | PROTEIN CHANGE | Hg19 nomenclature | Hg 37 nomenclature | Alternate allele | Allele count/total allele number | Reference |
| --- | --- | --- | --- | --- | --- | --- | --- |
| 1 | c.3276+1G>A | - | chr3:48624404 | chr3:48586971 | C>T alternate allele is G>A | 1/2054 | (Jain et al., 2021) |
| 2 | c.3565A>G | p.Met1189Val | chr3:48623665 | chr3:48586232 | T>C alternate allele is A>G | 1/5008 | (Jain et al., 2021) |
| 3 | c.6738G>T | p.Leu2246Phe | chr3:48610466 | chr3:48573033 | C>A alternate allele is G>T | 1/2048 | (Jain et al., 2021) |
| 4 | c.6082G>A | p.Gly2028Arg | chr3:48612870 | chr3:48575437 | CG>C alternate allele is G>A | 8/264882 | (Karczewski et al., 2020) |

References

Jain, A., Bhoyar, R. C., Pandhare, K., Mishra, A., Sharma, D., Imran, M., Senthivel, V., Divakar, M. K., Rophina, M., Jolly, B., Batra, A., Sharma, S., Siwach, S., Jadhao, A. G., Palande, N. V., Jha, G. N., Ashrafi, N., Mishra, P. K., Vidhya, A. K., … Sivasubbu, S. (2021). IndiGenomes: A comprehensive resource of genetic variants from over 1000 Indian genomes. *Nucleic Acids Research*, *49*(D1), D1225–D1232. https://doi.org/10.1093/nar/gkaa923

Karczewski, K. J., Francioli, L. C., Tiao, G., Cummings, B. B., Alföldi, J., Wang, Q., Collins, R. L., Laricchia, K. M., Ganna, A., Birnbaum, D. P., Gauthier, L. D., Brand, H., Solomonson, M., Watts, N. A., Rhodes, D., Singer-Berk, M., England, E. M., Seaby, E. G., Kosmicki, J. A., … Daly, M. J. (2020). The mutational constraint spectrum quantified from variation in 141,456 humans. *Nature*, *581*(7809), 434–443. https://doi.org/10.1038/s41586-020-2308-7
