# Supplementary material for: Clinical and allelic heterogeneity in dystrophic epidermolysis bullosa- lessons from an Indian cohort
Source: PLoS One. 2023 Aug 9;18(8):e0289558. doi: 10.1371/journal.pone.0289558 (PMC10411825; doi:10.1371/journal.pone.0289558)
Supplement: S1 File — (DOCX) [file pone.0289558.s004.docx]

**DNA EXTRACTION FROM BLOOD USING PHENOL-CHLOROFORM METHOD**

**Protocol**

1. Invert the vacutainer containing sample to mix well. Take 1ml of the blood sample in a 5ml centrifuge tube.
2. Add equal volumes of Lysis buffer 1 and incubate the tube at 65^0^C for 5 minutes.
3. Centrifuge the tube at 10000 rpm for 10 minutes at 14^0^C.
4. Retain the pellet and discard the supernatant which contains the cell debris.
5. To the pellet add equal volumes of Lysis buffer 2 and vortex the pellet.
6. Add 5µl of proteinase K per mL (dip tip in the solution and release).
7. Add 100µl of Sodium dodecyl sulfate(SDS) per mL.
8. Incubate at 56^0^C for 2 hours.
9. Add equal volumes of chilled Tris saturated phenol(pH 7.8) and mix thoroughly.
10. Centrifuge the lysate at 10,000 rpm for 10 minutes (15^0^C).
11. Collect the supernatant in a separate tube and add 1:1 ratio of Tris phenol to Chloroform: Isoamyl alcohol and mix thoroughly.
12. Centrifuge the lysate at 10,000rpm for 10 minutes (*following post centrifugation, the mixture separates as 3 layers; an upper colorless aqueous phase: white interphase: lower reddish organic phase*)
13. To the supernatant add equal volumes of chloroform: Isoamyl alcohol and mix it well.
14. Centrifuge the tube at 10,000rpm for 10 minutes (14^0^C).
15. Collect the supernatant in 5ml eppendorf tube and add 35µl (1/30 volume) of 3M Sodium acetate (pH 5.2).
16. Add double the volume of chilled ethanol. (*Ethanol precipitates out the DNA*)
17. Precipitate the DNA by swirling the tube few times.
18. Transfer the visible DNA into 1.5 mL tube. (Label on tube the patient details and secure with tape)
19. Centrifuge the tube at 10000rpm for 2 minutes.
20. Discard the supernatant and retain the pellet.
21. To the pellet add 500µl of 70% ethanol(for wash).
22. Centrifuge the tube at 10000rpm for 2 minutes.
23. Discard the ethanol and retain the pellet.
24. Air dry the pellet until all the traces of ethanol have evaporated (for 30 min or more).
25. Dissolve the pellet using nuclease free water (30-40µl depending on size of pellet).
26. Incubate the tube overnight at room temperature for the DNA to dissolve properly.
27. Quantify DNA using Nano Drop the next day.
28. Finally store the DNA at -20^0^C.

**PCR Amplification conditions**

1. Denaturation:

Denaturation is carried out at 95˚C for 30 sec. The double strand DNA gets denatured to single strands

2. Annealing:

Annealing is carried out for 30 sec at Tm-5C but not exceeding 72˚C for Taq DNA polymerase During this step, the primers base pair or anneal with single stranded template DNAs. Therefore, annealing temperature (Ta) is set at 5˚C less than the melting temperature (Tm) of the primers.

3. Extension:

During this step, the primer is extended by adding complementary bases at its 3’ hydroxyl end. Extension is carried out at 72˚C for Taq DNA polymerase. Duration of extension step depends on the length of the PCR product to be amplified. Extension time can be set at the rate of 0.5-1.0 minute/kb for Taq DNA polymerase, 1.0-2.0 minutes/kb for Pfu DNA polymerase.

PCR Cycles: Three consecutive steps of denaturation-annealing-extension constitutes one PCR cycle. The extension product is again denatured to begin the next PCR cycle. Likewise, about 25 to 35 PCR cycles are carried out to get desired quantity of PCR product that can be visualized by agarose gel electrophoresis and ethidium bromide staining.

**PCR purification**

**Procedure**

 Take PB buffer 5 times the PCR product.

 Add the entire PCR product to the eppendorf and retropiptte the solution.

 Take the entire solution in the gravity columns.

 Centrifuge at 10,000 rpm for 2 minutes.

 Discard the components in the flow-through; place the gravity column in the same tube.

 Add 750μl of the PE buffer to the gravity column.

 Centrifuge at 10,000 rpm for 2 minutes.

 Discard the components in the flow-through; place the gravity column in the same tube.

 Centrifuge at 10,000 rpm for 1-2 minutes.

 Open the gravity column lid and leave it for air dry for 10 minutes, place the gravity column in the different eppendorf (1.5ml preferred).

 Check for the ethanol smell, if it does not exist add 20μl of EB buffer and leave it for 30- 45 minutes.

 Centrifuge at 10,000, rpm for 2 minutes and the collect the flow through.

 Check the concentration of the flow-through using a nano drop and store the fresh PCR product in 4°C.

**GEL ELECTROPHORESIS**

**Procedure**

 Measure 2 g of agarose.

 Mix the agarose powder with 100ml of 1X TAE buffer.

 Microwave for 1-3 min until the agarose is completely dissolved (but do not over boil the solution, as some of the buffer will evaporate and thus alter the final percentage of agarose in the gel. Many people prefer to microwave in pulses, swirling the flask occasionally as the solution heats up).

 Let agarose solution cool down to about 50 °C (about when you can comfortably keep your hand on the flask), about 5 minutes.

 Add ethidium bromide (EtBr). EtBr binds to the DNA and allows you to visualize the DNA under ultraviolet (UV) light.

 Once solidified, place the agarose gel into the gel box (electrophoresis unit).

 Fill gel box with 1xTAE (or TBE) until the gel is covered.

 Carefully load a molecular weight ladder into the first lane of the gel. When loading the sample in the well, maintain positive pressure on the sample to prevent bubbles or buffer from entering the tip. Place the very top of the tip of the pipette into the buffer just above the well. Very slowly and steadily, push the sample out and watch as the sample fills the well. After the entire sample is unloaded, push the pipette to the second stop and carefully raise the pipette straight out of the buffer.

 Add loading buffer to each of your DNA samples. Loading buffer serves two purposes: 1) it provides a visible dye that helps with gel loading and allows you to gauge how far the DNA has migrated; 2) it contains a high percentage of glycerol that increases the density of your DNA sample causing it settle to the bottom of the gel well, instead of diffusing in the buffer.

 Carefully load your samples into the additional wells of the gel.

 Run the gel at 80-150 V until the dye line is approximately 75-80% of the way down the gel. Black is negative, red is positive. The DNA is negatively charged and will run towards the positive electrode

 Using any device that has UV light, visualize your DNA fragments. The fragments of DNA are usually referred to as ‘bands’ due to their appearance on the gel.

**Variant calling**

**Genomic DNA --- Library Preparation**

**Exome selection probes**



**Enriched regions**

**Custom bait capture system**



**Sequencing (100x) coverage**

**Paired end sequencing**



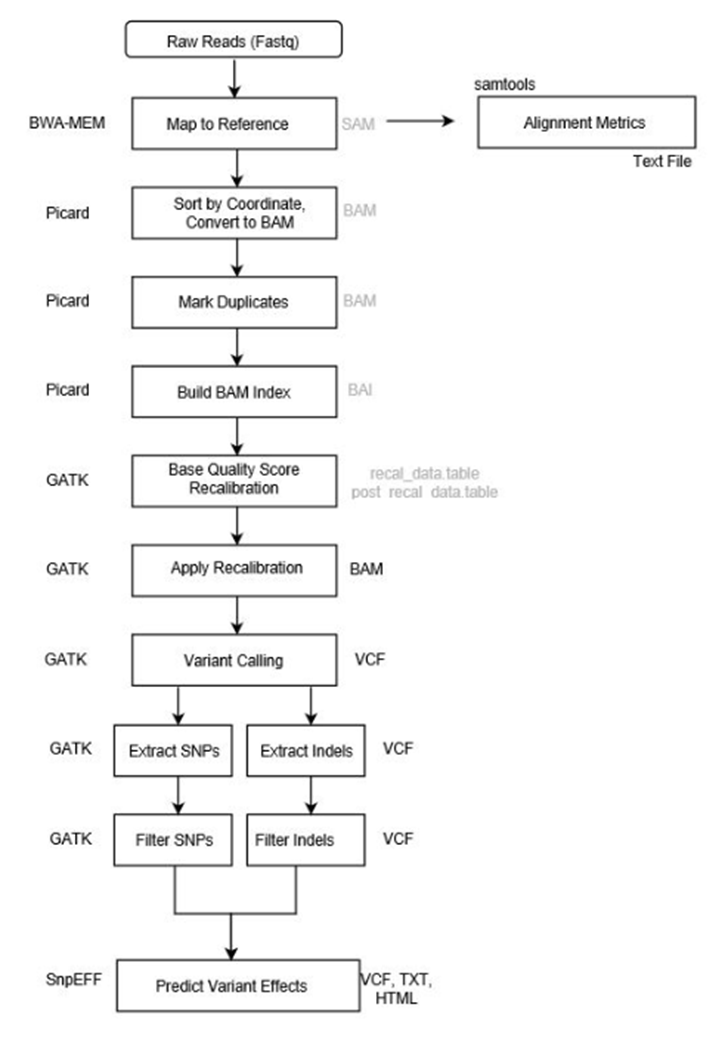


**Variant Annotation and Filtration**



Classification based on ACMG guidelines

**Report the Variant**

**Protocols for skin biopsy and immunofluorescence antigen mapping are similar to what we have used previously** (Hiremagalore R, Kubba A, Bansel S, Jerajani H. Immunofluorescence mapping in inherited epidermolysis bullosa: a study of 86 cases from India. Br J Dermatol. 2015;172:384-91)

**Protocol for skin biopsy:**

- Select a site: next to normal-looking skin close to a previously affected area or an active blister.
- The area is marked with a marker and rubbed gently with the thumb or a pencil eraser to induce a blister
- 2% lidocaine without adrenaline is injected to induce local anaesthesia.
- Elliptical skin biopsy is taken from the site
- Sample is transported in Michel’s medium for immunofluorescence antigen mapping.
- Punch biopsy is avoided to prevent shearing of the epidermis.

**Protocol for immunofluorescence antigen mapping:**

- Skin samples are washed in phosphate-buffered saline (PBS) for 1 h then snap frozen in liquid-nitrogen-cooled n-hexane.
- The lesional tissue and frozen normal human skin tissue are cut into 4-μm sections in a cryostat set at 25 °C, placed side by side on the same slide.
- Sections are fan dried for 10 min, washed in PBS solution for 30 min and rinsed in distilled water.
- After fan drying, the sections are incubated with primary antisera.
- The slides are placed in a lidded moist chamber, incubated for 45 min at 37 °C, washed in PBS solution for 30 min, rinsed in distilled water and fan dried for 10 min.
- Sections are covered with secondary antisera: polyclonal rabbit antimouse immunoglobulin/ fluorescein isothiocyanate rabbit F(abʹ)2 (Dako, Glostrup, Denmark) at 1 : 200 dilution.
- Slides are placed in a moist chamber, covered, incubated for 45 min at 37 °C, washed in PBS for 30 min, rinsed in distilled water and fan dried for 10 min.
- Sections are mounted with glycerol-based mounting medium for viewing.
- Slides are viewed using a Nikon Eclipse E200 Fluorescent microscope (Nikon, Tokyo, Japan).
- The primary antibodies used for the diagnosis and sub-typing of EB are against cytokeratin 14, laminin 332 (formerly known as laminin 5), type VII collagen and type IV collagen.

The sources of these antibodies are as follows:

| **Monoclonal antibody** | **Clone and supplier** | **Dilution** |
| --- | --- | --- |
| Mouse antihuman  cytokeratin 14 | LLOO2; AbD Serotec | 1 : 1000 |
| Mouse antihuman  laminin (laminin 5) | GB3; AbD Serotec | 1 : 100 |
| Mouse anticollagen  type VII | LH7.2 ascites fluid; Sigma | 1 : 300 |
| Mouse anticollagen  type IV | Col-94 ascites fluid; Sigma | 1 : 500 |

AbD Serotec, Oxford U.K.; Sigma, St Louis, MO, U.S.A.

## Sanger Sequencing

## Cycle sequencing protocol

1.Arrange and vortex and spin all the samples and primers

2.Take out RR mix from the coolant

3.Prepare master mix /cycle sequence cocktail

4.Add 18.5 µl of Master mix [18.5-18.9 µl ]

5.Add 0.2 µl of primers

6.Add template [50-100ng]

7.Briefly centrifuge for program 2[130g-1min]

8.Run for cycle sequencing program on veriti DX[Big-dye -kit-std]

9.Store at 4°C [If processed for next]

Clean up protocol

Master Mix 1: 20µl milliQ water and 4µl EDTA[125mM]

Master Mix 2 : 100 µl Absolute ethanol and 4.8 µl NaOAc

1. Prepare Master Mix 1 and 2 and label them
2. Add 24µl of master mix 1
3. Place septa on it and invert mix for 10-15 minutes.
4. Add 10 µl of Master mix
5. Place septa and invert mix for 40-50 times
6. Incubate for 15 minutes at room temperature
7. Spin the plate for program 1
8. Make plate record [2210g -30min]
9. Invert it gently on tissue paper and invert spin for program 2 [130g-1 min]
10. Add 200 µl of 70% ethanol
11. Place septa and spin the plate for program 3
12. Invert gently on tissue paper and invert spin for program 2[130g-1min]
13. Add 10 µl of foramide and brief spin for program 2[130g-1 min]
14. Denature by veriti and snap chill
15. Run on machine
